# Evaluating Ecological and Economic Benefits of a Low-Carbon Industrial Park Based on Millennium Ecosystem Assessment Framework

**DOI:** 10.1100/2012/909317

**Published:** 2012-12-18

**Authors:** Bin Chen, Guoxuan He, Jin Yang, Jieru Zhang, Meirong Su, Jing Qi

**Affiliations:** State Key Joint Laboratory of Environmental Simulation and Pollution Control, School of Environment, Beijing Normal University, Beijing 100875, China

## Abstract

The Millennium Ecosystem Assessment (MA) framework was modified with a special focus on ecosystem service values. A case study of a typical low-carbon industrial park in Beijing was conducted to assess the ecological and economic benefits. The total economic value of this industrial park per year is estimated to be 1.37 × 10^8^ RMB yuan, where the accommodating and social cultural services are the largest two contributors. Due to the construction of small grasslands or green roofs, considerable environmental regulation services are also provided by the park. However, compared with an ecoindustrial park, carbon mitigation is the most prominent service for the low-carbon industrial park. It can be concluded that low-carbon industrial park construction is an efficacious way to achieve coordinated development of society, economy, and environment, and a promising approach to achieving energy saving and carbon reduction.

## 1. Introduction

The services of ecological systems and the natural capital stocks are critical to the functioning of the Earth's life-support system. They contribute to human welfare, both directly and indirectly, and thereby represent a part of the total economic value of the planet [1]. However, these services are not fully recognized by human societies. The economic evaluation of ecosystem services is becoming an effective way to understand the multiple benefits provided by ecosystem services. Assessing the economic values of ecosystem services is thus an effective way to link human activities and natural systems [2]. As a specific interdisciplinary field of practice, ecosystem service evaluation has been conducted by many researchers. Daily provided a detailed compendium on describing, measuring, and valuing ecosystem services [3]. Costanza performed a monetary study to value the world's ecosystem service and natural capital [1] and further, conducted a multi-scale study to assess biodiversity and ecosystem service [4]. Hougner et al. investigated the economic valuation of a seed dispersal service in the Stockholm National Urban Park by taking into account biodiversity values [5]. Sattout et al. applied contingent valuation method to assess the economic value of cedar relics [6]. Spash et al. discussed the motives behind willingness to pay for biodiversity improvement in a water ecosystem [7]. However, these existing studies are still focused on estimating the value of natural ecosystem services with few studies valuing the artificial ecosystem services.

Artificial ecosystem services are similar to natural ecosystem services in essence, but differ in the following main aspects: (1) enhancement of certain services and decline of most other services in artificial ecosystems compared to natural ecosystems [8]; (2) higher direct use values than indirect use values are usually estimated through artificial ecosystem services in comparison with natural ecosystem services. Efforts have been made to estimate the ecosystem services of artificial systems, for example, Tian and Cai evaluated the ecosystem services of artificial landscapes in Beijing [9], and Shen et al. assessed the environmental and economic values for constructed wetlands [10]. 

Industrial park is a typical artificial ecosystem and functions as a small “city” with complete infrastructural facilities, internal material and information flows, and semi-artificial environmental conditions. As a new kind of industrial agglomeration mode, low-carbon industrial parks have recently been playing a key role in achieving global carbon emission mitigation and promoting a low-carbon economy. Low-carbon industrial park can be defined as a well-operated cluster of firms and organizations designed to maximize its social economic output and minimize its greenhouse gas (GHG) emissions. The implication of low-carbon parks does not only lie in energy conservation and emission reduction, but also a new mode featuring the integration of green, ecological and sustainable development, that is, practicing low-carbon ecological designs so as to realize the harmony between human society and nature.

The construction of low-carbon industrial parks can reduce GHG emissions and environmental pollution caused by energy consumption, thereby bringing in huge ecosystem service values. To quantify the ecosystem service provided by low-carbon industrial parks, the Millennium Ecosystem Assessment (MA) framework is employed in this paper. MA was initiated in 2001 with the objective to assess the consequences of ecosystem change for human well-being, which provided the scientific basis for actions needed to enhance the conservation and sustainable use of those systems. The involved works in the MA framework provide a state-of-the-art scientific appraisal of the condition and trends in the world's ecosystems and the services they provide (such as clean water, food, forest products, flood control, and natural resources) and the options to restore, conserve, or enhance the sustainable use of ecosystems.

 In this study, we proposed an ecosystem service evaluation framework based on MA to assess the ecological and economic value of low-carbon industrial parks.  Section 2 demonstrates the ecosystem services quantitation method. In Section 3, a case of a low-carbon industrial park in Beijing is introduced, and the results of ecosystem services evaluation are integrated and demonstrated. Finally, Section 4 presents the conclusions of this study.

## 2. Methodology

Potential ecosystem services brought by the low-carbon industrial parks are categorized into accommodating benefit, GHG emission reduction benefit, environmental benefit, and social benefit. Accommodating benefit results from the provision of workplace for the settled enterprises. GHG emission reduction benefit comes from the utilization of carbon-reducing building materials in the construction stage and application of renewable energy in the operation stage. Environmental benefit is derived from two approaches, that is, the utilization of renewable energy, which is beneficial in protecting forest resource and decreasing the traditional fossil energy consumption, and the green land project in the low-carbon industrial park. Social benefit is attributable to the provision of job opportunity.

**Figure 1:**
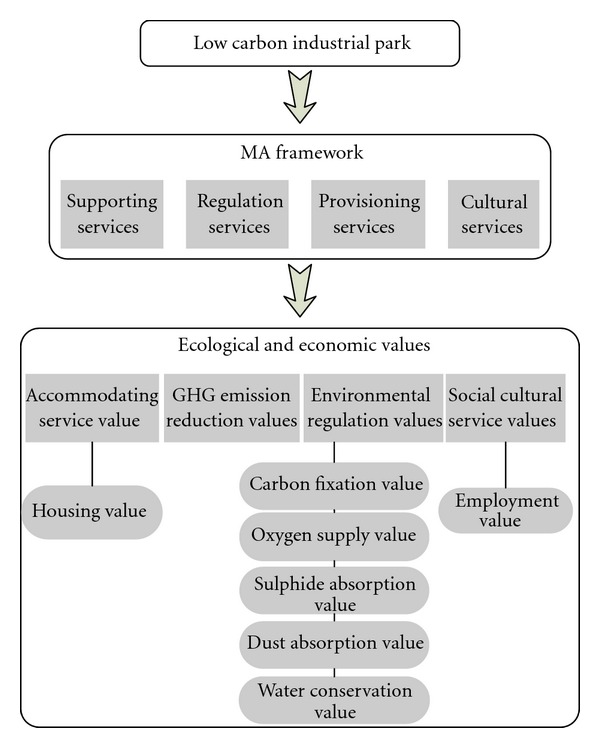
Ecological and economic value assessment framework of the low-carbon industrial parks.

In the MA framework, ecosystem services are divided into four parts, that is, supporting services, regulation services, provisioning services, and cultural services [2]. As most of these services can be provided by the low-carbon industrial park, the MA framework is appropriate to quantify these benefits in the form of monetary value. In this study, the ecosystem services of the MA framework are thereby quantified as four categories, that is, accommodating service value, GHG emission reduction value, environmental regulation value, and social cultural service value (Figure 1).

### 2.1. Accommodating Service Values

Low-carbon industrial park has accommodated many establishments and provided them with workspaces, so it generates a housing value (HV), which can be estimated by

(1) $$\begin{array}{c} \text{HV}=P_{H}\times \frac{A_{H}}{T_{H}}, \end{array}$$

where HV is the living value. *P*_*H*_ is the average selling price of buildings in the industrial park. *A*_*H*_ is the sales area. *T*_*H*_ is the years that land can be used by the industrial park.

### 2.2. GHG Emission Reduction Values (GV)

Comparing the carbon emissions of the concerned low-carbon industrial park with other traditional industrial parks, the carbon benefits of the low-carbon industrial park can be obtained. Combined with economic methods such as replacement cost or shadow price, the GHG emission reduction service value is calculated based on the price element in the Carbon Market Europe:

(2) $$\begin{array}{c} \text{GV}=P_{C}\times Q_{C}, \end{array}$$

where GV is GHG emission reduction value, *P*_*C*_ is the price of carbon in the CER Carbon Market Europe [11], and *Q*_*C*_ is the carbon emission reductions, in unit of tCO_2_ equivalent.

### 2.3. Environmental Regulation Values (ERV)

Based on the example of European Union's research about ecological economic values of small grasslands or green roof [12], the replacement cost method [13, 14] is employed to calculate the ecosystem services values of grassland and plants in low-carbon industrial parks.

#### 2.3.1. Carbon Fixation and Oxygen Supply Values (CV and OV)

CV can be estimated based on the photosynthesis equations and the shadow price of forestry restoration cost (20 dollars/t) [15]. The equation is shown as follows:

(3) $$\begin{array}{c} \text{CV}=T_{C}\times Q_{\text{C}{\text{O}}_{2}}\times 0.27, \end{array}$$

where CV is carbon fixation value, *T*_*C*_ is the shadow price of forestry restoration cost, *Q*_CO_2__ is the amount of CO_2_ fixation, and 0.27 is the carbon content coefficient of CO_2_ emission.

OV can be estimated by replacement cost method based on the Net Primary Productivity (NPP) and the cost of industrial oxygen production [15]. The equation is demonstrated below

(4) $$\begin{array}{c} \text{OV}=C_{{\text{O}}_{2}}\times \text{NPP}\times 1.2, \end{array}$$

where OV is the oxygen supply value, *C*_O_2__ is the cost of industrial oxygen production, NPP is the net primary productivity of plants, and 1.2 is oxygen release ratio per biomass production that is determined by the photosynthesis equation.

#### 2.3.2. Air Purification Values

In this study, the purification services of ecosystem mainly include the absorption of sulphide and dust [16], which are calculated as follows:

(5) $$\begin{array}{c} \text{SV}=C_{S}\times Q_{\text{S}{\text{O}}_{2}},\\ \text{DV}=C_{D}\times Q_{D}, \end{array}$$

where SV is the sulphide absorption value, *C*_*S*_ is the per unit SO_2_ reduction cost, derived from the average investment on SO_2_ treatment per unit SO_2_ reduction according the environmental statistics [17], and *Q*_SO_2__ is the amount of SO_2_ absorption; DV is dust absorption value, *C*_*D*_ is the cost of industrial de-dusting, and *Q*_*D*_ is the amount of dust absorption [15].

#### 2.3.3. Water Conservation Values (WV)

Water area in low-carbon industrial park provides the water conservation services, which can be estimated by shadow price approach using reservoir storage cost in China (0.67 RMB/m^3^) [16]. The equation is shown as follows:

(6) $$\begin{array}{c} \text{WV}=C_{W}\times Q_{W}, \end{array}$$

where WV is water conservation value, *C*_*W*_ is the per unit reservoir storage cost in China, and *Q*_*W*_ is the amount of water conservation in the industrial park.

Finally, environmental regulation values can be described as

(7) $$\begin{array}{c} \text{ERV}=\text{CV}+\text{OV}+\text{SV}+\text{DV}+\text{WV}. \end{array}$$

### 2.4. Social Cultural Service Values (SCV)

Low-carbon industrial park can provide multiple social cultural services, such as scientific research, production, landscape appreciation, and entertainment. Only the employment value is considered in this study due to data availability.

The low-carbon industrial park provides various employment opportunities, so the employment value can be estimated as follows:

(8) $$\begin{array}{c} \text{EV}=W_{3}\times F_{3}, \end{array}$$

where EV is employment value, *W*_3_ is the average wage per capita in tertiary industry, and *F*_3_ is the amount of employment in tertiary industry. 

## 3. Case Study

The concerned low-carbon industrial park is located in the northeast of Daxing District in Beijing. The industrial park has a floor space of 0.174 million square meters and built up area of 0.336 million square meters. Totally 43 buildings are distributed in this industrial park with high-end industries like intelligent, innovative, and design enterprises. So far, this industrial park has accommodated 49 enterprises with employment surpassing 2000 people.

The park covers a green area of 41,839 square meters and a water area of 5,073 square meters, of which the floor area ratio is 0.77 and the greening ratio is 41%. It emits only 0.7 ton CO_2_ equivalent per 10,000 GDP, which is less than 1/4 of that of the industrial output in China and 1/2 of that of the tertiary industrial [18]. A rainwater collection system and a waste water treatment system are also installed as auxiliary engineering to achieve water recycling and reuse.

Data for the low-carbon industrial park are provided by Beijing Development Area [18] including the total GHG emission per year, economic and environmental data, and information of settled enterprises. Some of the parameters are summarized and listed in Table 1.

We estimated each type of value in the low-carbon industrial park per year. The total ecological and economic value of the low-carbon industrial park is 1.37 × 10^8^ RMB yuan/a. It consists of accommodating service value, GV, ERV, and SCV, which are 1.13 × 10^7^, 1.07 × 10^6^, 2.31 × 10^6^, and 1.22 × 10^8^ RMB yuan/a, respectively. Specific values of each service are shown in Table 1.

**Figure 2:**
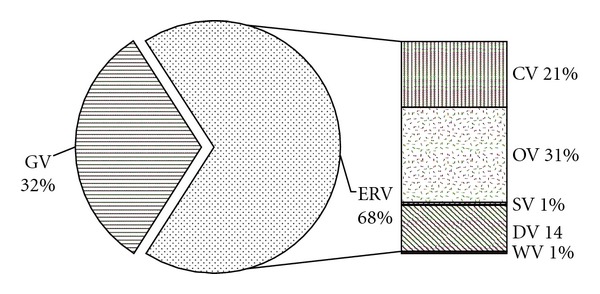
The proportion of CV and ERV of the low-carbon industrial park.

As shown in Table 2, we can see that the employment value (EV) accounts for the largest proportion of the total values with a percentage of nearly 89%. This is generally due to the type of companies in the industrial park, most of which belong to the tertiary industry with a much higher average salary level. Housing value also represents a large portion of the total value with a ratio of 8.24%. As the prices of house keep soaring, even residents with a national average salary cannot afford a house in Beijing, implying that the housing value of buildings is far more than its real value. These two values are the direct values that we can find in the market. However, the most precious values of low-carbon industrial park are environmental and ecological values that cannot be evaluated by market prices. Thus, a specific analysis of GV and ERV is made in the following part. The results are shown in Figure 2.

The total GV and ERV of the park is 3.38 × 10^6^ RMB yuan/a, which implies that 3.38 × 10^6^ RMB yuan more ecological benefits per year are gained in the park compared with traditional industrial park due to the utilization of renewable energy and green land construction. As shown in Figure 2, ERV constitutes the largest proportion of 68%, followed by the GV (32%). 

The constitutions of the ecological services are further decomposed in Figure 2. Among ERVs, the CV and OV make up the largest proportion. The DV also accounts for a significant portion of 14%. The SV value and WV only make up a relatively small fraction, indicating that the effects of low-carbon industrial park in SO_2_ emission reduction and water conservation are not prominent.

It can be seen that GV is only half of ERV, implying that low-carbon industrial parks are also a kind of ecoindustrial parks with emphasis on energy saving and emission reduction.

**Figure 3:**
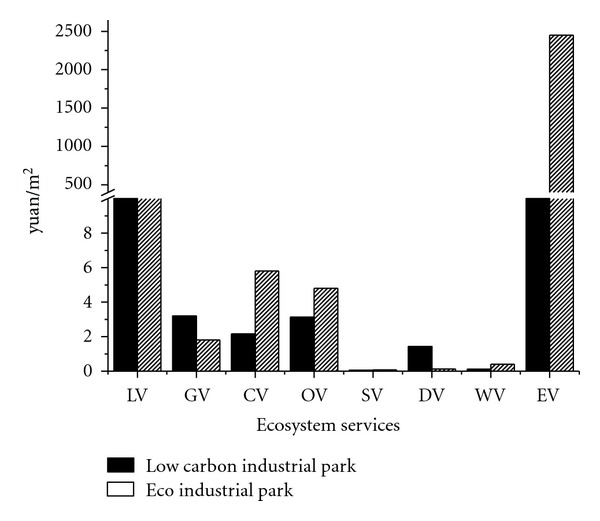
Comparison of the low-carbon industrial park with an ecoindustrial park.

The comparisons of this low-carbon industrial park and an ecoindustrial park [19] are demonstrated in Figure 3. The indicator of ecosystem service per area is used as a numéraire for the assessment. Obviously, the GV of the park is 1.77 times of that of the ecoindustrial park. However, the ERV of the ecoindustrial park (10.81) is a little higher than that of the low-carbon industrial park (6.86). Thus, we can conclude that the advantage of low-carbon industrial parks is carbon mitigation, rather than ecological construction, which is the theme of an ecoindustrial park. 

## 4. Conclusions

The total value of a low-carbon industrial park in Beijing is calculated to be 1.37 × 10^8^ RMB yuan/a. The results show that the low-carbon mode can bring the industrial parks tremendous ecological, social, and GHG benefits, especially in terms of ecosystem service and GHG emission reduction. This case is thus a benchmark for future industrial park construction in the context of low-carbon development. In addition, except for GHG emission, low-carbon industrial parks are of significant ecological importance. Therefore, the low-carbon industrial park is not only an effective measure to solve the contradiction between high-speed development and high emission in the economic society of industrial park, but also an efficacious way to achieve society-economy-ecology sustainable development. 

## Figures and Tables

**Table 1:** Main parameters of the low carbon industrial park.

Parameters	Value	Parameters	Value
Floor area	1.74*E* + 05 m^2^	Greening area	4.18*E* + 04 m^2^
Employment	2000	Water recycling rate	50.80%
Operation years	48 years	Registered capital	1.31*E* + 10 yuan
GHG emission intensity	0.07 tCO_2_ eq/yuan	GHG emission avoided	0.24 tCO_2_ eq/yuan

**Table 2:** The economic value of each ecological service.

Type	Service	Value (RMB yuan/a)
Accommodating service values	Housing value (HV)	1.13 × 10^7^
GHG emission reduction values (GV)	GHG emission reduction	1.07 × 10^6^
Environmental regulation values (ERV)	Carbon fixation (CV)	7.20 × 10^5^
	Oxygen supply (OV)	1.05 × 10^6^
	Sulfide absorption (SV)	2.10 × 10^4^
	Dust absorption (DV)	4.80 × 10^5^
	Water conservation (WV)	3.40 × 10^4^
Social cultural service values	Employment value (EV)	1.22 × 10^8^
Total values (SCV)		1.37 × 10^8^
